# A longitudinal study of the association between basal ganglia volumes and psychomotor symptoms in subjects with late life depression undergoing ECT

**DOI:** 10.1038/s41398-021-01314-w

**Published:** 2021-04-01

**Authors:** M. G. A. Van Cauwenberge, F. Bouckaert, K. Vansteelandt, C. Adamson, F. L. De Winter, P. Sienaert, J. Van den Stock, A. Dols, D. Rhebergen, M. L. Stek, L. Emsell, M. Vandenbulcke

**Affiliations:** 1 Neuropsychiatry, Department of Neurosciences, Leuven Brain Institute, Leuven, Belgium; 2 Department of Neurology, University Hospitals Leuven, Leuven, Belgium; 3 Geriatric Psychiatry, University Psychiatric Center, KU Leuven, Leuven, Belgium; 4 Academic Center for ECT and Neuromodulation (AcCENT), University Psychiatric Center, KU Leuven, Leuven, Belgium; 5 Department of Developmental Imaging, Murdoch Children’s Research Institute, Victoria, Australia; 6 Department of Psychiatry, VUmc Amsterdam, Amsterdam, the Netherlands; 7 Department of Old Age Psychiatry, GGZinGeest Amsterdam, Amsterdam, the Netherlands; 8 Mental Health Care Institute GGZ Centraal, Amersfoort, the Netherlands; 9 KU Leuven, Department of Imaging and Pathology, Translational MRI, Leuven, Belgium

**Keywords:** Depression, Neuroscience, Predictive markers

## Abstract

Psychomotor dysfunction (PMD) is a core element and key contributor to disability in late life depression (LLD), which responds well to electroconvulsive therapy (ECT). The neurobiology of PMD and its response to ECT are not well understood. We hypothesized that PMD in LLD is associated with lower striatal volume, and that striatal volume increase following ECT explains PMD improvement. We analyzed data from a two-center prospective cohort study of 110 LLD subjects (>55 years) receiving ECT. Brain MRI and assessment of mood, cognition, and PMD was performed 1 week before, 1 week after, and 6 months after ECT. Volumetry of the caudate nucleus, putamen, globus pallidus, and nucleus accumbens was derived from automatically segmented brain MRIs using Freesurfer®. Linear multiple regression analyses were used to study associations between basal ganglia volume and PMD. Brain MRI was available for 66 patients 1 week post ECT and in 22 patients also six months post ECT. Baseline PMD was associated with a smaller left caudate nucleus. One week after ECT, PMD improved and volume increases were detected bilaterally in the caudate nucleus and putamen, and in the right nucleus accumbens. Improved PMD after ECT did not relate to the significant volume increases in these structures, but was predicted by a nonsignificant volume change in the right globus pallidus. No volume differences were detected 6 months after ECT, compared to baseline. Although PMD is related to lower striatal volume in LLD, ECT-induced increase of striatal volume does not explain PMD improvement.

## Introduction

Depression is the most common cause of years lived with disability in Europe and a top ten cause of impaired health worldwide^1^. Primarily a disorder of mood, depression is also associated with cognitive and motor symptoms^2,3^. The term psychomotor dysfunction (PMD) is used in neuropsychiatry as a joint term for reduced and increased movement^4,5^. It involves the quality and quantity of gross and fine movements, facial expression, eye movement, gait, and posture^2,6,7^. The prevalence of PMD in major depressive disorder (MDD) ranges from 20% by clinical assessment to 60% by experimental testing^2^, and is higher in late life depression (LLD)^3^. It impairs activities of daily living, hinders therapy participation, and predicts chronicity of LLD^8^. In addition, PMD is a core feature of the melancholic depression subtype^9,10^, which responds well to tricyclic antidepressants and electroconvulsive therapy (ECT)^2,10–12^. Brain imaging of MDD patients with PMD has revealed metabolic, functional, and structural changes within the fronto-striatal network, basal ganglia, and supplementary-motor area (for review see refs. ^6,13–16^). Structural changes are reported most consistently in fronto-striatal regions and include subcortical white matter lesions (WML)^17,18^ as well as lower gray matter volume (GMV)^19–23^ (for review see refs. ^24–26^). Disease affecting the basal ganglia is known to produce mood, cognitive, and behavioral symptoms^5,7,27,28^. The symptom overlap between disorders of mood and movement suggests a shared pathway, which may have therapeutic implications. For instance, improvement of mood and movement after ECT has been reported for both MDD^29^ and Parkinson’s disease^30–32^. The neurobiology underlying the therapeutic effect of ECT remains unresolved. Recently, there has been increased focus on GMV increases following ECT, with consistent findings in the hippocampus, amygdala, and anterior cingulate cortex (for review see refs. ^33,34^). Volume increases after ECT have also been observed in the basal ganglia^22,33,35,36^, yet only one study analyzed its relation with PMD (post hoc). Using voxel-based morphometry (VBM), the study showed a correlation between volume increases in the caudate nucleus and PMD improvement^22^.

Our study investigated whether ECT-induced modulation of neuro-anatomic substrates of PMD is related to clinical outcome. We analyzed a large sample of subjects to test the hypothesis that LLD patients with pronounced PMD have lower volumes of specific basal ganglia structures, that these volumes increase after a course of ECT, and that these volume increases are associated with improvement of PMD. We analyzed brain imaging and clinical data collected 1 week before (baseline, *t*_0_), 1 week after (*t*_1_) and 6 months after ECT (*t*_2_)^37^. Four regions of interest (ROI) were chosen based on previous literature and involvement in PMD in LLD^14–16,20,36–39^: the caudate nucleus, the putamen, the globus pallidus, and the nucleus accumbens.

## Materials and methods

### Subject inclusion and exclusion

**Fig. 1:**
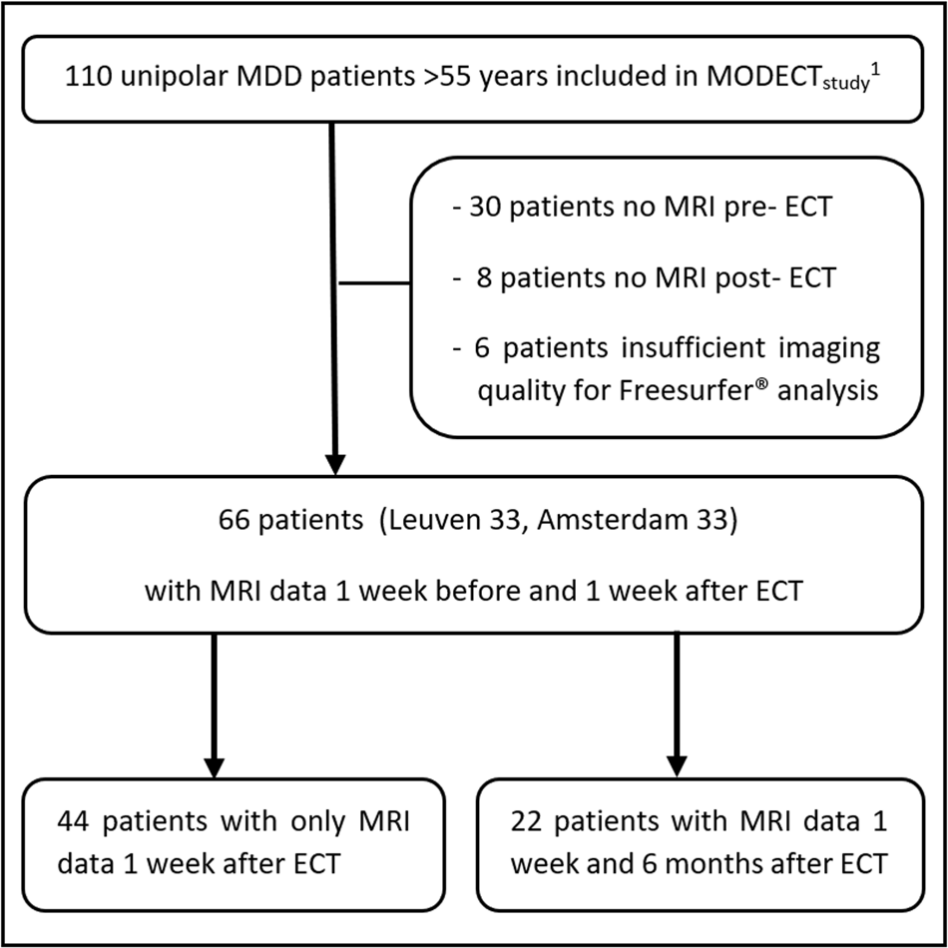
Flowchart of inclusion/exclusion process. ^1^MODECT Mood Disorders in Elderly treated with ECT study (Dols et al.^37^), MDD major depressive disorder, ECT electroconvulsive therapy, MRI magnetic resonance imaging.

Data were obtained from the Mood Disorders in Elderly treated with ECT study (MODECT)^37^. Patients were included at the University Psychiatric Center KU Leuven, Leuven (Belgium) and GGZ inGeest, Amsterdam (the Netherlands) between January 1^st^ 2011 and December 31^st^ 2013. Screening and enrollment was performed by a psychiatrist. Inclusion criteria were age ≥55 years, major unipolar depression according to the Diagnostic and Statistical Manual of Mental Disorders (DSM) IV criteria (ed. 2000), indication for ECT during the recruitment period. Primary indications for ECT were pharmacotherapy resistance, life-threatening symptoms, elective, or other. Exclusion criteria were a DSM IV diagnosis of bipolar or schizoaffective disorder or a major neurological illness. The MODECT study included 110 patients (67 from Amsterdam). We excluded 44 patients (34 from Amsterdam) not meeting the quality criteria for automated volumetric analysis of T1 MRI data (due to movement artefacts or premature scan abort) or with missing MRI data on *t*_0_ or *t*_1_ (Fig. 1), which reduced the sample to a total of 66 included participants. The study protocol was approved by the ethical review board of the University Hospitals Leuven and VU University Medical Center. Participant’s written informed consent was obtained at study entry. The study was conducted according to the declaration of Helsinki and registered at www.ClinicalTrials.gov with identifier: NCT02667353.

### Clinical and epidemiological data

Demographic and clinical variables including comorbidity and medication use were obtained by a semi-structured interview and checked by chart review. The diagnosis of depression was based on the DSM-IV criteria, using the Mini-International Neuropsychiatric Interview (M.I.N.I., ed. 1998). Antidepressant treatment was assessed with the Antidepressant Treatment History Form (ATHF). Clinical outcome variables were psychomotor function, measured with the CORE (total and subscales), global cognitive functioning quantified with the Mini-Mental State Examination (MMSE, ed. 1975) score and depressive symptoms, measured with the Montgomery Åsberg Depression Rating Scale (MADRS, ed. 1979) score. Trained research nurses and psychologists blinded to clinical information, performed the scoring. The CORE rating scale (referring to “core features” of melancholia) was used to quantify PMD^4,40^. This observer-dependent scale comprises 18 items rated on a four-point scale, “0” indicating absence, “1” slight, “2” moderate, and “3” marked severity of a symptom. The score includes three subscores that reflect three dimensions of PMD: the non-interactiveness (6 items), motor retardation (7 items), and motor agitation (5 items) subscore. High inter-observer reliability has been demonstrated with intra-class correlation coefficients of 0.80 to 0.87^41^. A total CORE score ≥8 was used to define the melancholic depression subtype^41^.

### Intervention

After study entry, patients received ECT twice weekly, according to standardized guidelines^42^. The decision to perform ECT was made by two experienced psychiatrists according to local guidelines. Psychotropic medication was discontinued at least 1 week prior to ECT, or if impossible, kept stable 6 weeks prior to and during ECT. An ECT course started with right unilateral brief pulse ECT (RUL-ECT) in all but 2 patients who started with bilateral ECT (BL-ECT). Brief pulse ECT (0.5–1.0 ms) was administered with the Thymatron System IV (maximum energy 200%, 1008 mCoulombs). The stimulus intensity was determined by empirical dose titration at the first treatment, for RUL-ECT 6 times the initial seizure threshold (ST) and for BL-ECT 1.5 times the ST. Motor seizures of less than 20 s were considered inadequate and followed by dose increase according to standardized guidelines^42^. Weekly clinical evaluations were performed by a psychiatrist, who decided on the number of ECT sessions and switch to BL-ECT. A switch to BL-ECT was performed in case of aggravating clinical condition or failed improvement after 6 unilateral treatments. Criteria for clinical worsening were: increased MADRS-score, debilitating psychotic features, increased suicidality, dehydration, weight loss. ECT was either continued until a MADRS score <10 was achieved at two consecutive ratings with a 7-day interval, or stopped after a minimum of 6 unilateral and 6 bilateral sessions if no further clinical improvement (measured with the MADRS) had occurred in the last 2 weeks of ECT.

### Image acquisition and processing

**Fig. 2:**
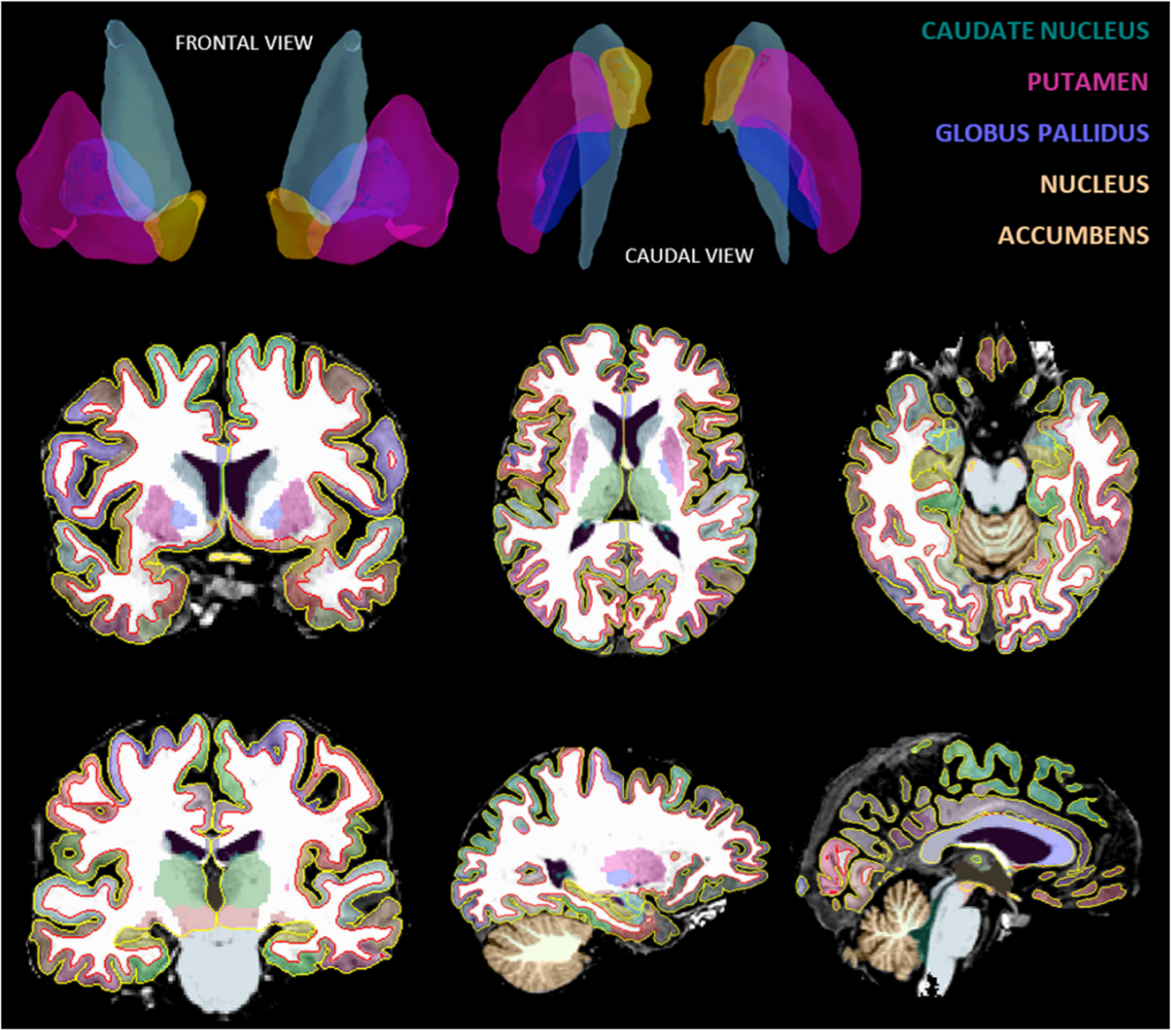
Volumetric 3D segmentation of high-resolution 3D T1-weighted MR images using Freesurfer 6.0.0 (Fisher et al. 2012). Caudate nucleus, putamen, globus pallidus, and nucleus accumbens volumes were extracted from whole-brain segmentation images. Participants were scanned at three time points: prior to ECT (baseline, *t*_0_), one week after (*t*_1_), and six months after ECT completion (*t*_2_). The volumetric data were normalized to account for differences in total brain volume using a standard approach (Jack et al.^48^).

High-resolution 3D T1-weighted images were acquired using an eight-channel head-coil with a 3D turbo field echo sequence on a 3T Philips Intera Scanner in Leuven and 3T GE Signa HDxt scanner in Amsterdam (TR = 9.6 ms, TE = 4.6 ms, flip angle = 8°, slice thickness = 1.2 mm, in-plane voxel size = 0.98 x 0.98 x 1.2 mm^3^, 182 slices, acquisition time = 383 s). Participants were scanned at three time points: prior to ECT (*t*_0_), one week after (*t*_1_), and six months after ECT completion (*t*_2_). T1 images were pre-processed with bias correction using N4ITK^43^, then processed with Freesurfer® 6.0.0 (Fig. 2). Subcortical segmentation and volumetric quantification with Freesurfer® is documented and freely available online (http://surfer.nmr.mgh.harvard.edu/); technical details are described in prior publications^44–47^. In a small number of cases, voxels containing dura mater were included in the white matter mask. These were removed by masking out voxels within 3 mm of the brain boundary using binary erosion of the brain mask. For subjects with multiple timepoints, the standard longitudinal pipeline^46^ was applied to perform refinement of cross-sectional results. Caudate nucleus, putamen, globus pallidus, and nucleus accumbens GMV were taken from whole-brain segmentation images. The volumetric data was normalized to account for differences in total brain volume using a standard approach^48^. Imaging outcome variables were GMV (mm^3^) of the four basal ganglia ROI described above.

### Data analysis

#### Analysis 1: cross-sectional analysis at baseline (*t*_0_)

Clinical and demographic outcome differences between study sites were analyzed with an independent Student’s *t* test or, if non-normally distributed, with a Mann–Whitney U test in case of continuous variables. For categorical variables, chi-square tests were used. Baseline imaging predictors of psychomotor function (PMD, dependent variable, measured with the total CORE score) were identified using multiple linear regression analysis with each basal ganglia ROI volume as the independent variable of interest and age, sex, MADRS score, and study site as co-variates. Compliance with regression assumptions (linearity, homoscedasticity, normal distribution of residuals, no multicollinearity) was verified for each regression analysis. Separate analyses were done including bilateral (sum scores of left and right) and unilateral volumes of each ROI as the independent variable of interest. In addition, we performed a subscale analysis focusing on the CORE retardation and also the agitation subscale, though retardation is considered a more stable feature of melancholic depression as compared to agitation and has shown a stronger correlation to instrumental measurements of movement^49^.

#### Analysis 2 and 3: longitudinal analysis

We first studied the volume alteration of basal ganglia ROI (ΔROI) and the difference in total CORE score (ΔCORE) after ECT separately (analysis 2), using paired Student’s *t* tests for the time interval between *t*_1_ and *t*_0_ (early effects), as well as between *t*_2_ and *t*_0_ (late effects). Second, we investigated the relationship between ΔCORE and ΔROI as a result of the ECT course (analysis 3). For this analysis, a linear multiple regression analysis similar to analysis 1 was performed, with ΔCORE as the dependent variable and one of the four basal ganglia ΔROI and the number of administered ECT’s (#ECT’s) as the independent variables of interest. Age, sex, total CORE baseline score, and study site were co-variates. Similar to analysis 1, we also replaced the total CORE score by the CORE retardation subscale and by the agitation subscale in a separate model. Lastly, to study the effects of ECT duration and electrode placement on ΔROI, we also performed multiple linear regression analyses with each unilateral ΔROI as the dependent variable and #ECT’s, electrode placement (RUL- or BL-ECT), age, sex, and site as covariates.

Statistical analysis was conducted with IBM SPSS statistical software (SPSS, version 25, SPSS Inc., Chicago, IL). A *p*-value of 0.05, 2-sided, was considered statistically significant. Bonferroni–Holm correction was performed to correct for multiple comparisons. For the regression analyses, Bonferroni–Holm correction was based on the overall *F* test in order to select significant models for each set of regression models. Univariate outliers of normalized ROI data were addressed as standardized values outside the absolute value of 3.29 (extreme outlier) in all analyses containing imaging data^50^.

## Results

### Patient characteristics

Patient characteristics (*n* = 66; Table 1 (selection), Table S1 (full)), were comparable between study sites, except for a higher CORE retardation subscale (3 [1;6] vs 1 [0;2], *p* = 0.020) and higher prevalence of melancholic depression (91 vs 65%, *p* = 0.011) in Leuven (*n* = 33) and a more frequent switch to bilateral ECT in Amsterdam (42 vs 15%, *p* = 0.006)^37^. Results of the MMSE and MADRS have been published previously^29,37^. The total CORE score correlated with the MADRS (*r* = 0.417, *p* = 0.001) and MMSE (*r* = −0.241, *p* = 0.039).

**Table 1:** Patient characteristics.

	*n*(%)/ mean ± SD/ median [IQR]
Age (Yrs)	72 ± 8.4
Sex: female	43 (65%)
Site: Leuven	33 (50%)
Late onset depression	31 (47%)
Melancholic depression	49 (80%)
Psychotic depression	32 (49%)
MDD duration (months)	6 [3;12]
MADRS baseline	35 [29;41]
MMSE baseline	24 [21;28]
CORE total baseline	15 [8;22]
CORE non-interaction	5 [2;8]
CORE agitation	8 [4;10]
CORE retardation	2 [0;4]
Time *t*_0_ to ECT (weeks)	2 [1;5]
Medication during ECT	17 (32.1%)
ECT course duration (days)	39 [28;48]
Number of ECT sessions	11 [8;14]
Only RUL ECT	47 (71.2%)

*MADRS* Montgomery Åsberg Depression Scale, *n* = 64.

*MMSE* Mini-Mental State Examination, *n* = 57. The CORE rating scale, *n* = 61.

*MDD duration* duration of current major depression episode, *n* = 61.

Time *t*_0_ to ECT = time between baseline MRI and ECT start, *n* = 62.

*ECT* electroconvulsive therapy, *RUL* right unilateral.

### Psychomotor symptoms and basal ganglia volume at baseline

**Fig. 3:**
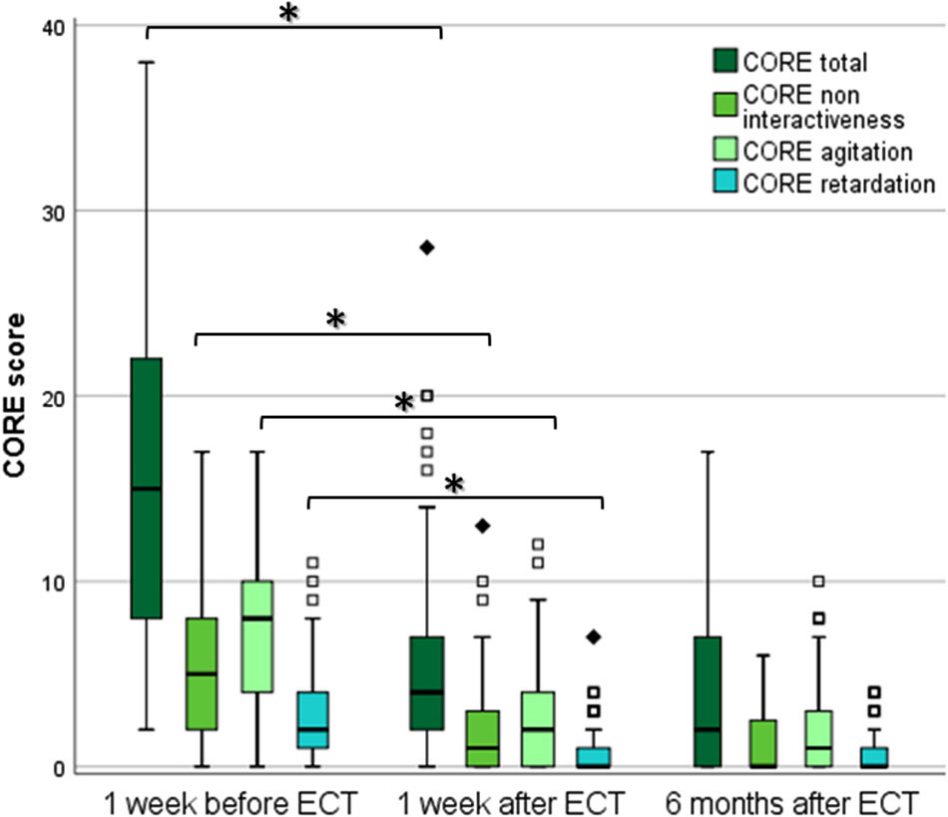
CORE total and CORE subscale scores before and after ECT. *ECT* electroconvulsive therapy. *p < 0.05, Bonferroni–Holm correction applied. Black squares = outliers (>|2x SD|), diamonds = extreme outliers (>|3x SD|).

Psychomotor symptoms (PMD) were measured at baseline (*t*_0_) and 1 week post ECT (*t*_1_) in 61 patients (Fig. 3, Table 1). The median total CORE score of the sample was 15 [8,22]. Inter-subscale correlation was significant for the CORE non-interactiveness and CORE agitation subscale (*r* = 0.776, *p* < 0.001), but not for the CORE retardation and the agitation (*r* = 0.008, *p* = 0.995) or non-interactiveness subscale (*r* = 0.263, *p* = 0.440). Patients had higher scores on the CORE agitation subscale (i.e., more agitation symptoms) compared to the CORE retardation subscale (Table 1). A smaller baseline volume of the caudate area predicted a higher total CORE score (*F*(5, 52) = 4.61, *β*^std^-0.265, *p* = 0.039) (Fig. S1, Table S2). Unilateral analysis revealed that the effect was attributed to the left caudate nucleus (*F*(5,53) = 4.88, *β*^std^ -0.346, *p* = 0.011). The baseline volumes of the putamen, globus pallidus, accumbens area, and the total intracranial volume did not significantly predict the total CORE score. Only in the unilateral analysis, a smaller left accumbens volume predicted a lower total CORE score (Table S2). The CORE retardation subscale score was significantly predicted by a smaller baseline caudate area (*F*(5,53) = 3.31; *β*^std^ -0.379, *p* = 0.006). In the unilateral analysis, both left (*F*(5,53) = 3.30; *β*^std^ -0.395, *p* = 0.006) and right caudate nucleus (*F*(5,53 = 3.22; *β*^std^ -0.373, *p* = 0.006) predicted the CORE retardation subscale score (i.e., smaller volumes predicted a higher score). The CORE agitation subscale was not predicted by the volumes of the caudate nucleus, the putamen, globus pallidus, or accumbens nucleus.

### Evolution of psychomotor symptoms and basal ganglia volume one week after ECT

**Fig. 4:**
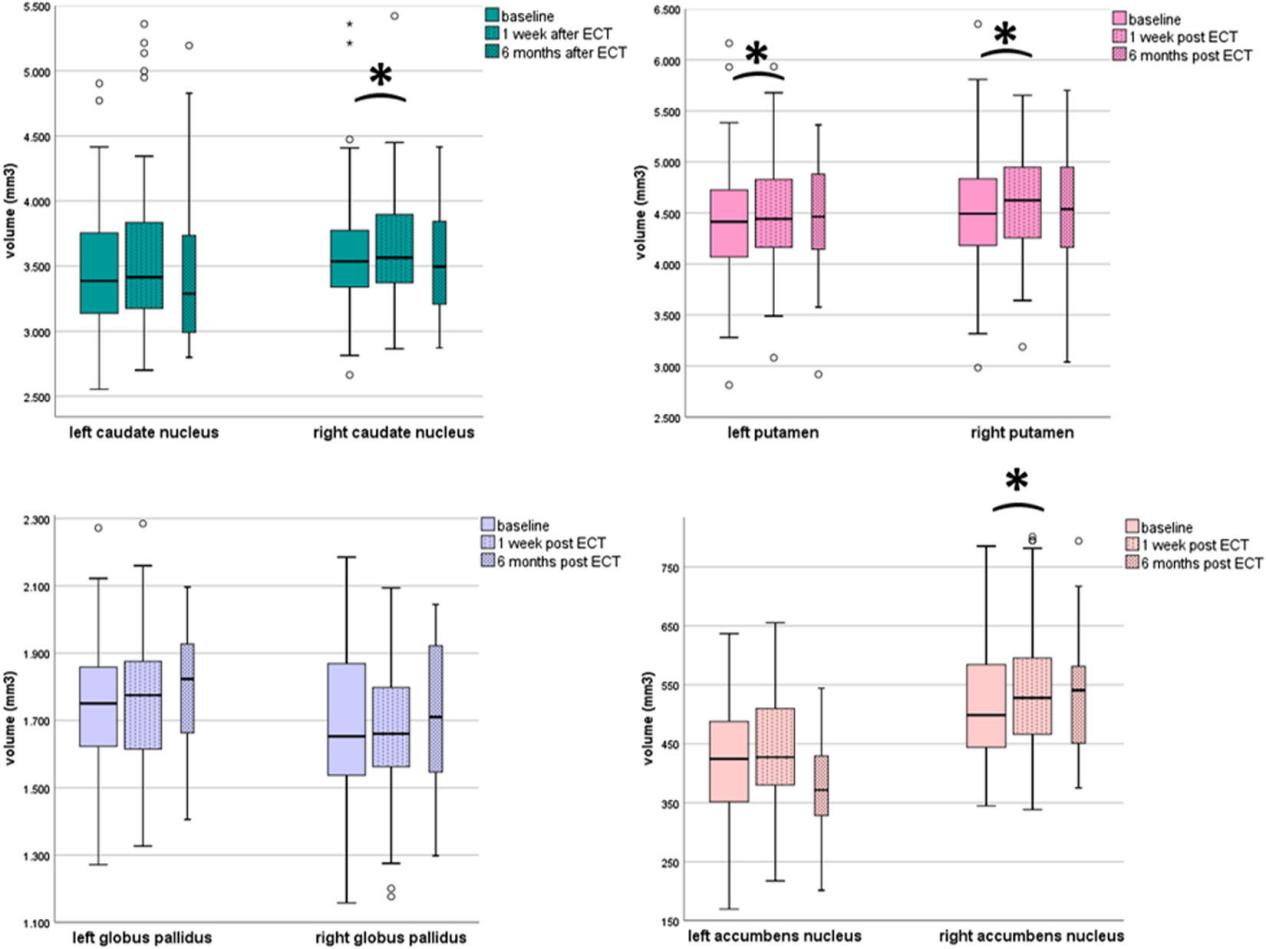
Basal ganglia volumes before and after ECT. Box-and-Whisker’s plot of basal ganglia ROI volume at baseline (*t*_0_, *n* = 66), one week after (*t*_1_, *n* = 66), and six months after ECT completion (*t*_2_, *n* = 22). Student’s paired *t* test for related samples, sign. level *α* = 0.05 (2-tailed), *p significance adjusted after Bonferroni–Holm correction for multiple testing. Black squares = outliers (>|2x SD|), diamonds = extreme outliers (>|3x SD|).

A robust improvement of the total CORE score and all subscale scores was observed after ECT (Fig. 3), as reported previously^29^. The total CORE score decreased with an average of 11.2 points (±9.41; *p* < 0.001), the retardation score with 2.1 (±2.67; *p* < 0.001), the agitation score with 4.9 (±4.40; *p* < 0.001) and the non-interactiveness score with 4.2 points (±4.73; *p* < 0.001). Patients with a melancholic depression (total CORE score ≥ 8 points) received more ECT sessions, (13 ± 5.2 vs 10 ± 7.8, *F* = 3.892, *p* < 0.001). One week after ECT, GMV increases were observed bilaterally for the caudate nucleus, putamen, and right accumbens nucleus (Fig. 4, Table S3), though the increase of the left caudate nucleus was not statistically significant after Bonferroni–Holm correction. The largest GMV increases occurred in the right accumbens nucleus (4.9%, *p* < 0.001), the putamen (right 2.6%, *p* < 0.001, left 2.3%, *p* < 0.001), and the right caudate nucleus (1.6%, *p* = 0.011). The observed volume increases were not significantly predicted by electrode placement (data not shown). Only the volume increase of the left caudate nucleus was predicted by the number of ECT’s (*F*(5,53) = 5.719; *β*^std^ 0.434, *p* < 0.001) and by the total CORE change (*F*(5,53) = 5.719; *β*^std^ -0.345, *p* = 0.005). The improvement in the total CORE score after ECT was not predicted by the observed volume increases of the caudate nucleus, putamen, or right accumbens nucleus (Table S4). Not restricting to regions with a significant volume increase after ECT, however, the volume change of the right globus pallidus predicted the improvement in the total CORE score (*F*(6,54) = 43.119, *β*^std^-0.127, *p* = 0.035). The subscale analysis showed that this effect only involved the CORE agitation subscale (*F*(6,53) = 25.947, *β*^std^-0.149, *p* = 0.041). Other significant predictors of improvement of the total CORE score were the baseline total CORE score (all models) and number of ECT’s (model with left or right caudate) (Table S4). Adding electrode placement as a covariate to these models did not influence the results (data not shown).

### Psychomotor symptoms and basal ganglia volume 6 months after ECT

The improvement of the total CORE score and its subscale scores persisted 6 months after ECT (*t*_2_) (Fig. 3). The median change between *t*_2_ and baseline for the total CORE score was 12.7 points (±10.28; *p* < 0.001), for the retardation score 2.1 points (±2.85; *p* < 0.001), for the agitation score 5.7 (±4.89; *p* < 0.001), and for the non-interactiveness score 5.0 (±5.00; *p* < 0.001). Volumetric analysis of the ROI at *t*_2_ was available for 22 patients (Fig. 4, Table S3). One patient was excluded from *analysis 3* because of maintenance ECT after *t*_1_. Compared to baseline, no volumetric differences in the basal ganglia ROIs were observed at six months after Bonferroni–Holm correction (Table S3). We therefore did not perform further analyses at six months.

## Discussion

In a large cohort of LLD patients, we found (1) that PMD was related to lower gray matter volume in the striatum, (2) that ECT induced a transient increase in striatal gray matter volume, and (3) that this volume increase was not related to PMD improvement after ECT.

### PMD is correlated with reduced striatal GMV

Focusing on GMV differences between LLD patients with varying severity of PMD, our study showed that patients with more PMD have reduced GMV of the left caudate nucleus, independent of the severity of the depression. Focusing only on psychomotor retardation, both left and right caudate nucleus volumes were reduced in patients with a high CORE retardation subscale score. Healthy aging studies have demonstrated that smaller volumes of the striatum^51^ and globus pallidus^52^ are directly related to slowing of gait (for review see ref. ^53^) and that age-related reduced functional connectivity in the striatum impairs motor function^54^. However, atrophy of the dorsal striatum has also been linked to neurodegenerative movement disorders such as Parkinson’s disease^55,56^ and Huntington’s disease^57^. Structural alterations of the basal ganglia in MDD have been observed in several studies, most consistently a lower volume of the caudate nucleus^20,21,58–61^ and putamen^19,20,58^. Nuclear imaging studies have demonstrated reduced presynaptic dopamine transporter (DAT)^39,62^ and postsynaptic dopamine 1 receptor (D1-R) binding^63,64^ as well as reduced dopaminergic signaling^38^ in the basal ganglia of MDD patients. Functional MRI studies found reduced responsivity of the dorsal striatum and aberrant frontostriatal connectivity^65–67^. Only one study related imaging results of MDD patients to PMD, and found a correlation between smaller caudate nucleus and slowing on the trail making test as an unspecific measure for PMD^21^. Taken together, our findings in LLD patients support the contribution of the striatum to (psycho)motor dysfunction in aging and depression. Of note, only the CORE retardation subscale score (and not the non-interaction or agitation subscale score) was correlated with a smaller caudate nucleus volume bilaterally. Two recent studies demonstrated that the CORE retardation subscale, but not the agitation subscale, correlates well with instrumental motor assessment^49,68^. The results of the CORE retardation subscale score may therefore be more reliable.

### Striatal volume is increased one week after ECT

Our study found GMV increases of about 2–4% in the putamen (bilaterally), right caudate nucleus, and right accumbens nucleus one week after ECT. This is in line with the 2.4% volume gain of the left putamen observed by Wade et al. in the first week after 10 ECT sessions^36^, as well as the recent GEMRIC consortium meta-analysis (including data from refs. ^22,36^) that reported widespread GMV increases after ECT in both hemispheres^33^. The latter study reported higher effect sizes for subcortical regions including the caudate nucleus and putamen, that were mediated by the number of ECT sessions and increased towards the electrode placement side^33^. Our study showed an association between the number of ECT sessions and GMV increase in the caudate nucleus, but not electrode placement, similarly to the findings of Wade et al.^36^. Two hypotheses dominate the debate on the pathophysiology of ECT-induced GMV increase (for review see ref. ^69^). The first assumes that volume increases reflect cytotoxic edema^70–72^. However, the absence of cortical edema post-ECT in three studies, evaluated with either fluid-attenuated inversion recovery (FLAIR) MRI, T2 relaxation time or DWI, contradicts this^73–75^. Moreover, seizures may cause transient edema in cortical, superior juxtacortical or hippocampal regions, but not typically in the striatum^76^. The second "neuroplasticity" hypothesis comes from observations in the rodent hippocampus^77–79^ and subventricular zone (SVZ). Neuroblast migration from the SVZ into the striatum can be evoked by electroconvulsive shock (ECS)^80,81^ and prolonged seizure^82^. In adult humans, the dentate gyrus of the hippocampus and the lateral ventricle wall (SVZ equivalent) are known sites for neurogenesis. From the lateral ventricle wall, neuroblasts habitually migrate rostrally to become striatal interneurons^83^, which paves a theoretical path for ECT-induced striatal neuroplasticity similar to ECS in animals. In addition to structural alterations, PET research provides functional support to the neuroplasticity hypothesis. Increased postsynaptic D1-R transmission in the striatum, substantia nigra, and accumbens nucleus appears shortly after ECS in healthy animals^84–88^ and hemi-parkinsonian rats^89^, and striatal postsynaptic D3-R binding has been reported to increase after ECS in rodents^89,90^. Conceptually, enhanced striatal dopamine release after ECT could upregulate postsynaptic dopaminergic receptors in the direct dopaminergic pathway that facilitates movement. It is tempting to connect the increase in striatal dopaminergic transmission after ECT to the GMV alterations of the striatum and improvement of movement. However, the temporal evolution of structural and biochemical (dopaminergic) alterations after ECT reveals important dissimilarities. In concordance with studies investigating long-term effects of ECT on hippocampal GMV^35,73^, we found that basal ganglia GMV declined to baseline six months after ECT, although psychomotor outcome remained unchanged. Leaver et al. observed how the GMV increase in the caudate nuclei returned to baseline shortly after ECT, but the cerebral blood flow (CBF) continued to increase up to six months in the caudate and globus pallidus^35^. A waning of basal ganglia changes following ECS has also been observed in PET animal studies, with dopaminergic activity returning to baseline between 8 days to 6 weeks after ECS^86,87^. This hints at different temporal effects of ECT on clinical, structural, and neurotransmitter alterations, which should be accounted for in future study designs.

### Striatal GMV increase is not significantly related to psychomotor improvement after ECT

Contrary to our a priori hypothesis, PMD improvement did not correlate with the observed ECT-induced striatal GMV changes after ECT. In a voxel-based morphometry study, our group previously reported a correlation between ECT-induced GMV increase of the caudate nucleus and psychomotor improvement on the total CORE scale^22^. Nevertheless, the analysis was performed post-hoc on a smaller sample, did not include other basal ganglia structures (i.e., striatal output nuclei), and did not apply a correction for covariates in its statistical approach. Applying a different covariate-controlled statistical approach in a larger sample, this study shows no relation between ECT-induced GMV increase of the striatum and improved psychomotor function one week after ECT. However, we observed that volume change of the right globus pallidus, a striatal output structure that did not show a statistically significant volume increase after ECT, was related to PMD improvement. This finding is difficult to interpret, since the right pallidal volumetric changes equally comprised volume increases and decreases, suggesting an inconsistent ECT effect. Furthermore, this structure has not been classically linked to PMD in literature, warranting further caution. Nevertheless, this is a novel finding, that merits further exploration if a statistical type 2 error is ruled out by replication.

The lack of a clinical correlate of ECT-induced striatal volume increases is in line with studies that found no or even negative relationships between GMV change, mainly in the hippocampus, and mood or cognitive outcome. Two studies reported correlations of improved depression outcome (Hamilton Depression Rating Scale (HDRS), MADRS) with hippocampal volume increase after ECT^91,92^, but larger and pooled studies could not replicate this^33,73,93–97^. Studies evaluating cognitive function after ECT in relation to hippocampal volume increase revealed either no^34,98^ or a negative interaction^99,100^. Whether the clinical improvement of mood and psychomotor function after ECT is a direct or indirect effect of electric current stimulation, and how this relates to the upregulation of mono-aminergic neurotransmission discussed above, remains unclear. At 6 months post ECT, we measured no significant GMV differences in the basal ganglia as compared to baseline, notwithstanding a maintained clinical improvement of PMD. This suggests that the ECT effects on GMV may be transient. It is difficult to define whether ECT still affects clinical outcome at this time point, since other therapies such as antidepressant medication, psychotherapy, and psychomotor therapy may contribute to the sustained clinical improvement over the course of a six month interval.

### Limitations

Our study has important limitations. First, not all patients were medication free. During ECT, 17 patients (26%) were on psychotropic drugs such as benzodiazepines, antidepressants, mood stabilizers, and antipsychotics. After ECT termination, patients gradually started antidepressant therapy which generates accumulated therapeutic effects we could not control for at *t*_2_. Even though a recent pooled analysis showed no influence of drug status on brain volume alterations after ECT^33^, conclusions drawn from the imaging data at *t*_2_ should be taken with precaution, even more so given the relatively small sample size (*n* = 22) at this time point. Second, an inter-observer bias may result from data collection by study nurses, psychiatrists, and psychologists. The two-site design may also influence data collection. Although imaging was performed on similar devices with a uniform protocol, and Freesurfer®’s segmentation software is robust for multicenter scanning differences^45^, the Amsterdam sample displayed larger normalized volumes of several basal ganglia ROI, which we addressed for by implementing site as a covariable in all the regression analyses. Finally, the CORE scale may not be the most optimal measure of (psycho)motor function. The scale was developed to select patients with melancholic depression. Only a handful of studies validated it against an instrumental motor measure with moderate results^4,41,49,68^. Notwithstanding the high inter-comparator consistency, its observer-dependence and inclusion of affective and cognitive aspects may hinder an accurate PMD evaluation. Instruments such as the Unified Parkinson’s Disease Rating Scale part III (UPDRS III), experimental drawing tasks, or simple motor tasks (for review see ref. ^6^) may be more appropriate to assess motor dysfunction associated with basal ganglia dysfunction.

### Future directions

Future research should explore objective measures of PMD that better distinguish between ’pure’ motor, cognitive-executive, and affective-motivational aspects and relate this to the neuroanatomy of PMD before and after ECT. Second, the nature and cause of the GMV alterations after ECT should be investigated with advanced imaging techniques that allow structural, hemodynamic, and metabolic analyses of the brain. Image processing techniques such as shape analysis of striatal subregions as well as multimodal MRI could characterize the nature of the observed GMV alterations in the basal ganglia. Functional techniques offer another opportunity to explore GMV alterations in depth, such as PET-imaging tracers that measure synaptic density and accumulation of toxic proteins such as amyloid, tau, or alfa synuclein. By understanding the biochemical nature of the observed striatal volume alterations, we may gain insight not only into the effects of ECT on the basal ganglia, but also in the pathophysiology of PMD at a broader trans-nosological and neuro-anatomical level.

## Supplementary information

Table S1: patient characteristics and comparison Amsterdam vs Leuven

Table S2: multiple linear regression analysis of the CORE total at baseline (dependent) with the basal ganglia ROI, site, age, Sex and MADRS at baseline (*t*_0_).

Figure S1: scatterplot of the CORE total (A) and CORE retardation (B) score with the left caudate nucleus volume at baseline (*t*_0_).

Table S3: volumetric analysis of basal ganglia structures before (*t*_0_), 1 week after (*t*_1_) and 6 months after (*t*_2_) ECT

Table S4: multiple linear regression analysis of the difference of the total CORE score (dependent) with the volume difference in basal ganglia ROI, site, age, Sex, total CORE score baseline and the number of ECT’s as independent variables, between one week after ECT (*t*_1_) and baseline (*t*_0_).
