# Supplementary material for: A longitudinal study of the association between basal ganglia volumes and psychomotor symptoms in subjects with late life depression undergoing ECT
Source: Transl Psychiatry. 2021 Apr 1;11:199. doi: 10.1038/s41398-021-01314-w (PMC8017007; doi:10.1038/s41398-021-01314-w)
Supplement: Supplementary file 1 — Table S1: patient characteristics and comparison Amsterdam vs Leuven [file 41398_2021_1314_MOESM1_ESM.docx]

**Suppl. Table S1: patient characteristics and comparison Amsterdam vs Leuven**

|  | **Total (n=66)** n(%)/ mean ±SD/ median [IQR] | **Amsterdam (n=33)** n(%)/ mean ±SD/ median [IQR] | **Leuven (n=33)** n(%)/ mean ±SD/ median [IQR] | ***p*** |
| --- | --- | --- | --- | --- |
| Age (Yr) | 72 ± 8.4 | 72 ± 9.5 | 72 ± 7.5 | 0.974^1^ |
| Sex: female | 43 (65) | 20 (65 ) | 23 (66) | 0.919^2^ |
| N° Previous MDD episodes (n=59) | 3 [1; 4] | 3 [1; 5] | 3 [1; 4] | 0.657^3^ |
| LOD | 31 (47) | 13 (42) | 18 (51) | 0.441^2^ |
| Duration of current MDD episode before inclusion (months, n=61) | 6 [3; 12] | 8 [3; 17] | 6 [4; 8] | 0.339^3^ |
| MADRS baseline (n=64) | 35 [29; 41] | 34 [25; 43] | 35 [30; 38] | 0.656^3^ |
| MMSE baseline (n=57) | 24 [21; 28] | 25 [21; 28] | 24 [21; 28] | 0.922^3^ |
| CORE total baseline (n=61)  CORE non-interaction  CORE agitation  CORE retardation | 15 [8; 22]  5 [2; 8]  8 [4; 10]  2 [0; 4] | 13 [6; 21]  3 [1; 9]  7 [3; 10]  1 [0; 2] | 16 [11; 24]  5 [4; 8]  8 [6; 10]  3 [1; 6] | 0.098^3^  0.169^3^  0.603^3^  0.020^3^* |
| Melancholic depression (n=61) | 49 (80) | 17 (65) | 32 (91) | 0.011^2^* |
| Psychotic depression (n= 64) | 32 (49) | 16 (52) | 16 (48) | 0.632^2^ |
| Medication resistance (n=62) | 4 [2; 4] | 3 [2; 4] | 4 [3; 4] | 0.232^3^ |
| Time t_0_ MRI to ECT (n=62) (weeks) | 2 [1; 5] | 3 [1; 5] | 2 [1; 4] | 0.683^3^ |
| ECT course duration (days) | 39 [28; 48] | 39 [28; 53] | 39 [28; 45] | 0.364^3^ |
| N° ECT sessions | 11 [8; 14] | 11 [8; 16] | 11 [9; 14] | 0.969^3^ |
| Switch to BIL-ECT | 19 (29%) | 14 (42%) | 5 (15%) | 0.006^2^* |

^1^Student's T test, ^2^Chi2 Test, ^3^Mann-Witney U Test. LOD= late onset depression (>55 yrs). MDD= major depressive disorder. MADRS= Montgomery Åsberg Depression Scale, n=64. MMSE= Mini-Mental State Examination, n=57. The CORE rating scale, n=61. MDD duration= duration of current major depression episode, n=61. Time t_0_ to ECT= time between baseline MRI and ECT, n=62. ECT= electroconvulsive therapy. BIL-ECT= bilateral ECT stimulation.
