# Supplementary material for: A longitudinal study of the association between basal ganglia volumes and psychomotor symptoms in subjects with late life depression undergoing ECT
Source: Transl Psychiatry. 2021 Apr 1;11:199. doi: 10.1038/s41398-021-01314-w (PMC8017007; doi:10.1038/s41398-021-01314-w)
Supplement: Supplementary file 2 — Table S2: multiple linear regression analysis of the CORE total at baseline (dependent) with the basal ganglia ROI, site, age, Sex and MADRS at baseline (t0). [file 41398_2021_1314_MOESM2_ESM.docx]

**Suppl. Table S2: multiple linear regression analysis of the CORE total at baseline (dependent variable) with the ROI, site, age, sex and MADRS at baseline as independent variables.**

| **ROI** | **R^2^/R^adj^** | **F(5,x)** | **Site** | **Age** | **sex** | **MADRS** | **ROI** |
| --- | --- | --- | --- | --- | --- | --- | --- |
|  | | | ***Regression coefficients (Standard error)*** | | | | |
| **TIV** | 0.491/0.241 | F(5,55)=3.492** | 0.17 (2.345) | 0.06 (0.140) | 0.075 (2.815) | 0.428 (0.117)** | -0.072 (0.000) |
| **Caudate nucleus**  **Left**  **Right** | 0.307/0.241  0.315/0.251  0.270/0.201 | F(5,52)=4.611**  F(5,53)=4.882**  F(5,53)=3.919** | 0.08 (2.299)  0.06 (2.356)  0.10 (2.300) | 0.12 (0.135)  0.12 (0.134)  0.08 (0.138) | -0.01 (2.256)  -0.01 (2.249)  -0.003 (2.323) | 0.458 (0.117)***  0.456 (0.116)***  0.481 (0.119)*** | -0.265 (0.001)*  -0.346 (0.003)*  -0.143 (0.003) |
| **Putamen**  **Left**  **Right** | 0.236/0.166  0.238/0.169  0.240/0.169 | F(5,54)=3.344*  F(5,55)=8.436**  F(5,54)= 3.401* | 0.22 (2.223)  0.19 (2.222)  0.21 (2.243) | 0.92 (0.138)  0.07 (0.137)  0.10 (0.138) | 0.05 (2.289)  0.03 (2.291)  0.05 (2.276) | 0.403 (0.118)**  0.433 (0.117)**  0.403 (0.118)** | 0.022 (0.011)  0.021 (0.002)  0.061 (0.002) |
| **Globus pallidus**  **Left**  **Right** | 0.239/0.169  0.242/0.173  0.247/0.178 | F(5,55)=3.445**  F(5,55)=3.506**  F(5,55)=3.606** | 0.18 (2.261)  0.20 (2.212)  0.16 (2.298) | 0.07 (0.137)  0.07 (0.137)  0.07 (0.136) | 0.03 (2.327)  0.05 (2.330)  0.02 (2.298) | 0.438 (0.119)**  0.420 (0.119)**  0.448 (0.117)*** | -0.023 (0.003)  0.064 (0.006)  -0.101 (0.005) |
| **Accumbens**  **Left**  **Right** | 0.248/0.180  0.297/0.233  0.240/0.171 | F(5,55)=3.637**  F(5,55)=4.648**  F(5,55)=3.483** | 0.12 (2.645)  0.07 (2.373)  0.22 (2.598) | 0.13 (0.159)  0.23 (0.158)  0.05 (0.146) | 0.03 (2.259)  0.05 (2.189)  0.04 (2.277) | 0.433 (0.116)**  0.414 (0.112)**  0.431 (0.117)** | 0.137 (0.008)  0.317 (0.013)*  -0.061 (0.012) |

Linear multiple regression analysis, sign. level p <0.050*, p < 0.010**, p<0.001***. TIV = total intracranial volume. MADRS= Montgomery Åsberg Depression Scale. ROI= region of interest.
