# Supplementary figures and images for: A longitudinal study of the association between basal ganglia volumes and psychomotor symptoms in subjects with late life depression undergoing ECT
Source: Transl Psychiatry. 2021 Apr 1;11:199. doi: 10.1038/s41398-021-01314-w (PMC8017007; doi:10.1038/s41398-021-01314-w)

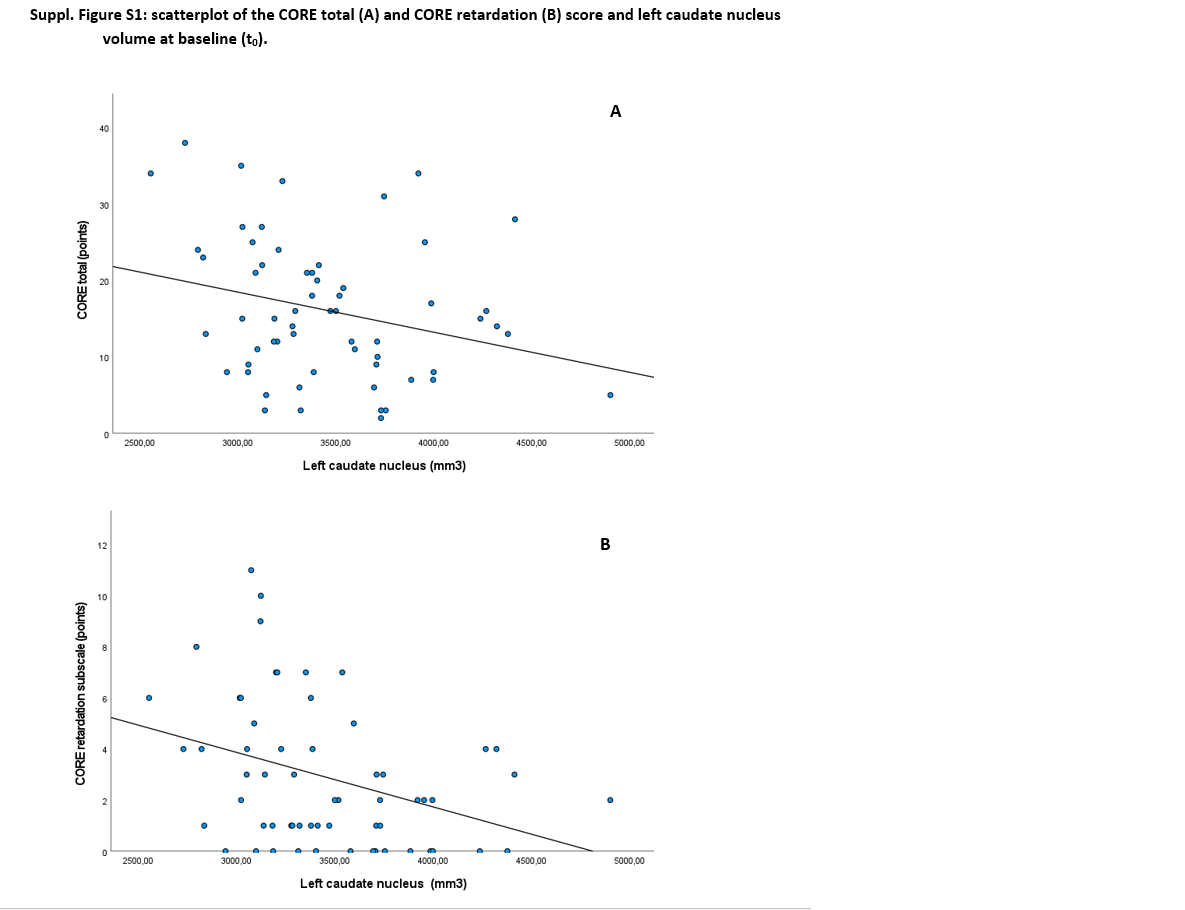

Supplement: Supplementary file 3 — Figure S1: scatterplot of the CORE total (A) and CORE retardation (B) score with the left caudate nucleus volume at baseline (t0). [file 41398_2021_1314_MOESM3_ESM.tif]
