# Supplementary material for: A longitudinal study of the association between basal ganglia volumes and psychomotor symptoms in subjects with late life depression undergoing ECT
Source: Transl Psychiatry. 2021 Apr 1;11:199. doi: 10.1038/s41398-021-01314-w (PMC8017007; doi:10.1038/s41398-021-01314-w)
Supplement: Supplementary file 4 — Table S3: volumetric analysis of basal ganglia structures before (t0), 1 week after (t1) and 6 months after (t2) ECT [file 41398_2021_1314_MOESM4_ESM.docx]

**Suppl. Table S3: volumetric analysis of basal ganglia structures before, 1 week after and 6 months after ECT**

|  | **Volume (mm^3^) ECT - 1W (n= 66)**  **mean ±SD** | **Volume (mm^3^)**  **ECT + 1W (n=66)**  **mean ±SD** | **Diff to baseline**  **ECT + 1W**  **median [IQR]** | **p^1^** | **Volume (mm^3^)**  **ECT+ 6M (n= 22)**  **mean ±SD** | **Diff to baseline**  **ECT+ 6M (n= 22)**  **median [IQR]** | **p^2^** |
| --- | --- | --- | --- | --- | --- | --- | --- |
| **Caudate**  **left**  **right** | 7009 ± 842.3  3492 ± 499.0  3565 ± 462.7 | 7130 ± 841.4  3541 ± 512.2  3624 ± 401.1 | *+112.1 [249.45]*  *+48.1 [180.82]*  *+59.2 [180.87]* | <0.001*  0.037  0.011* | 6754 ± 757.4  3391 ± 528.3  3481 ± 422.9 | *- 197.0 [587.30]*  *-129.5 [399.23]*  *-100.0 [386.70]* | 0.149  0.153  0.126 |
| **Putamen**  **left**  **right** | 8815 ± 986.7  4368 ± 549.8  4470 ± 503.1 | 9041 ± 988.2  4468 ± 535.9  4588 ± 491.8 | *+226.0 [268.60]*  *+100.2 [177.68]*  *+117.4 [161.86]* | <0.001*  <0.001*  <0.001* | 8908 ± 1182.4  4454 ± 626.4  4509 ± 580.6 | *+ 64.0 [323.50]*  *+19.6 [185.13]*  *-29.3 [174.71]* | 0.386  0.633  0.452 |
| **Glob pall**  **left**  **right** | 3398 ± 3405.6  1733 ± 200.8  1665 ± 215.4 | 936 ± 189.1  1744 ± 199.2  1661 ± 204.3 | *+7.1 [136.34]*  *+11.0 [73.86]*  *-3.8 [87.26]* | 0.672  0.231  0.721 | 3482 ± 391.2  1783 ± 193.5  1700 ± 218.8 | *+ 3.0 [147.00]*  *- 7.6 [105.42]*  *+10.3 [74.10]* | 0.935  0.737  0.523 |
| **Accumbens**  **left**  **right** | 936 ± 189.1  416 ± 102.0  519 ± 107.7 | 967 ± 188.1  422 ± 97.5  545 ± 114.9 | *+31.7 [66.81]*  *+6.0 [49.30]*  *+25.8 [51.80]* | <0.001*  0.328  <0.001* | 909 ± 173.6  377 ± 89.2  532 ± 107.4 | *- 9.0 [84.30]*  *- 28.1 [60.97]*  *+19.4 [62.15]* | 0.635  0.042  0.158 |

^1,2^ Student's T test for related samples, sign. level (2-tailed) α <0.05, *= significant after Bonferroni-Holm correction. ECT= electroconvulsive therapy. Diff= volume difference. W=weeks; M= months. SD= standard deviation. IQR= interquartile range.
