# Supplementary material for: A longitudinal study of the association between basal ganglia volumes and psychomotor symptoms in subjects with late life depression undergoing ECT
Source: Transl Psychiatry. 2021 Apr 1;11:199. doi: 10.1038/s41398-021-01314-w (PMC8017007; doi:10.1038/s41398-021-01314-w)
Supplement: Supplementary file 5 — Table S4: multiple linear regression analysis of the difference of the total CORE score (dependent) with the volume difference in basal ganglia ROI, site, age, Sex, total CORE score baseline and the number of ECT’s as independent variables, between one week after ECT (t1) and baseline (t0). [file 41398_2021_1314_MOESM5_ESM.docx]

**Suppl. Table S4: multiple linear regression analysis of the difference of the total CORE score (dependent) with the volume difference in ROI, site, age, Sex, total CORE score baseline and the number of ECT's as independent variables, between one week after ECT (T_1_) and baseline (T_0_).**

| **ROI** | **R^2^/R^adj^** | **F(6,X)** | **Site** | **Age** | **Sex** | **# ECT** | **CORE baseline** | **ΔROI** |
| --- | --- | --- | --- | --- | --- | --- | --- | --- |
|  | | | ***Regression coefficients (Standard error)*** | | | | | |
| **Caudate**  **left**  **right** | 0.806/0.783  0.811/0.790  0.804/0.781 | F(6,51)=35.330***  F(6,52)=37.277***  F(6,51)=34.791*** | -0.003 (1.223)  0.01 (1.198)  -0.002 (1.249) | -0.03 (0.075)  -0.04 (0.074)  -0.03 (0.075) | -0.08 (1.229)  -0.08 (1.202)  -0.07 (1.231) | 0.150 (0.124)*  0.167 (0.128)*  0.136 (0.120)* | -0.893 (0.072)***  -0.880 (0.070)***  -0.908 (0.069)*** | -0.059 (0.003)  -0.093 (0.005)  -0.016 (0.005) |
| **Putamen**  **left**  **right** | 0.811/0.789  0.806/0.784  0.817/0.796 | F(6,53)=37.824***  F(6,53)=36.791***  F(6,53)=39.366*** | -0.01 (1.198)  -0.01 (1.211)  -0.01 (1.179) | 0.01 (0.072)  -0.01 (0.074)  -0.002 (0.071) | -0.04 (1.227)  -0.06 (1.264)  -0.04 (1.173) | 0.100 (0.123)  0.123 (0.123)  0.095 (0.119) | -0.937 (0.069)***  -0.911 (0.074)***  -0.922 (0.065)*** | 0.077 (0.002)  -0.010 (0.004)  0.107 (0.003) |
| **Glob. pall.**  **left**  **right** | 0.824/0.804  0.903/0.815  0.910/0.827 | F (6,54)=42.036***  F(6,54)=38.640***  F (6,54)=43.119*** | 0.01 (1.148)  0.01 (1.174)  0.02 (1.137) | 0.001 (0.069)  0.007 (0.071)  -0.004 (0.069) | -0.53 (1.154)  -0.044 (1.182)  -0.063 (1.150) | 0.130 (0.115)*  0.131 (0.118)*  0.123 (0.114)* | -0.900 (0.064)***  -0.915 (0.065)***  -0.898 (0.063)*** | -0.110 (0.004)  -0.053 (0.008)  -0.127 (0.006)* |
| **Accumbens**  **left**  **right** | 0.811/0.790  0.813/0.792  0.814/0.793 | F(6,53)=37.900***  F(6,54)=39.083***  F(6,54)=38.770*** | 0.01 (1.158)  0.01 (1.182)  0.01 (1.177) | 0.01 (0.072)  0.01 (0.071)  0.01 (0.072) | -0.05 (1.212)  -0.05 (1.203)  -0.05 (1.117) | 0.125 (0.119)  0.119 (0.121)  0.111 (0.119) | -0.922 (0.065)***  -0.923 (0.065)***  -0.919 (0.064)*** | 0.028 (0.009)  -0.022 (0.013)  -0.066 (0.012) |

Linear multiple regression analysis, sign. level p <0.050 *, p < 0.010**, p<0.001***. #ECT= the number of ECT sessions administered. CORE baseline = total CORE score at baseline. ΔROI= the volume difference between basal ganglia ROI 1 week after ECT and baseline.
